# Cochlear protein biomarkers as potential sites for targeted inner ear drug delivery

**DOI:** 10.1007/s13346-019-00692-5

**Published:** 2019-11-18

**Authors:** James G. Naples, Lauren E. Miller, Andrew Ramsey, Daqing Li

**Affiliations:** 1 Division of Otolaryngology-Head and Neck Surgery, Beth Israel Deaconess Medical Center, 110 Francis St, Suite 6E, Boston, MA 02215 USA; 2 Department of Otorhinolaryngology-Head and Neck Surgery, Massachusetts Eye and Ear Infirmary, 243 Charles Street, Boston, MA 02114 USA; 3 Department of Otorhinolaryngology-Head and Neck Surgery, University of Pennsylvania School of Medicine, 421 Curie Blvd, Rm 1220, Philadelphia, PA 19104 USA; 4 Department of Otorhinolaryngology-Head and Neck Surgery, University of Pennsylvania Health System, 3400 Spruce St, 5 Silverstein, Philadelphia, PA 19104 USA

**Keywords:** Inner ear, Targeted-drug delivery, Biomarkers, Hearing loss

## Abstract

The delivery of therapies to the cochlea is notoriously challenging. It is an organ protected by a number of barriers that need to be overcome in the drug delivery process. Additionally, there are multiple sites of possible damage within the cochlea. Despite the many potential sites of damage, acquired otologic insults preferentially damage a single location. While progress has been made in techniques for inner ear drug delivery, the current techniques remain non-specific and our ability to deliver therapies in a cell-specific manner are limited. Fortunately, there are proteins specific to various cell-types within the cochlea (e.g., hair cells, spiral ganglion cells, stria vascularis) that function as biomarkers of site-specific damage. These protein biomarkers have potential to serve as targets for cell-specific inner ear drug delivery. In this manuscript, we review the concept of biomarkers and targeted- inner ear drug delivery and the well-characterized protein biomarkers within each of the locations of interest within the cochlea. Our review will focus on targeted drug delivery in the setting of acquired otologic insults (e.g., ototoxicity, noise-induce hearing loss). The goal is not to discuss therapies to treat acquired otologic insults, rather, to establish potential concepts of how to deliver therapies in a targeted, cell-specific manner. Based on our review, it is clear that future of inner ear drug delivery is a discipline filled with potential that will require collaborative efforts among clinicians and scientists to optimize treatment of otologic insults.

**Figure:**
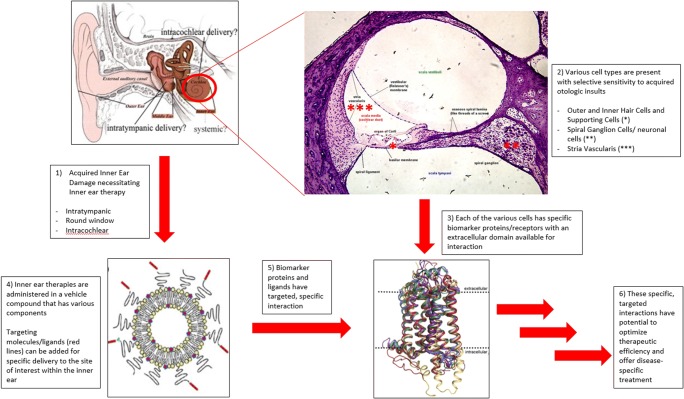
Graphical Abstract

Graphical Abstract

## Introduction

The cochlea is a privileged organ within the temporal bone that is protected from the external environment by the otic capsule bone, the round window membrane (RWM), and blood-labyrinth barrier. There are various cell types within the cochlea, which are particularly sensitive to disruptions of the local environment. Small changes within the local environment of the cochlea can introduce acquired insults, which present as a sensorineural hearing loss (SNHL) in the clinical setting and can be accompanied by tinnitus and vertigo. These characteristics introduce significant challenges when treating acquired cochlear disorders. There are various well-established etiologies of acquired cochlear insult that include excessive noise exposure, ototoxic medications, and idiopathic causes such as Meniere’s disease and sudden SNHL. While each of these various etiologies produces a similar clinical effect of SNHL, there is evidence to suggest that many of the causes of acquired SNHL preferentially introduce insults to specific locations within the cochlea (inner hair cells (IHC) [1], outer hair cells (OHC) [2], stria vascularis (SV) [3], spiral ganglion neurons (SG) [4], and supporting cells [5]). The specificity of ototoxicity is not managed differently in the clinical setting based on location of insult, and our current clinical therapies are non-specific. Despite these challenges, clinical and basic science research has led to early foundational understandings of the various mechanisms by which therapies can penetrate the protective barriers and reach locations within the cochlea [6]. However, the ability of treatments to target only the specific sites of insult remains elusive and is an area of active research that necessitates identification of target-specific biomarkers.

**Fig. 1:**
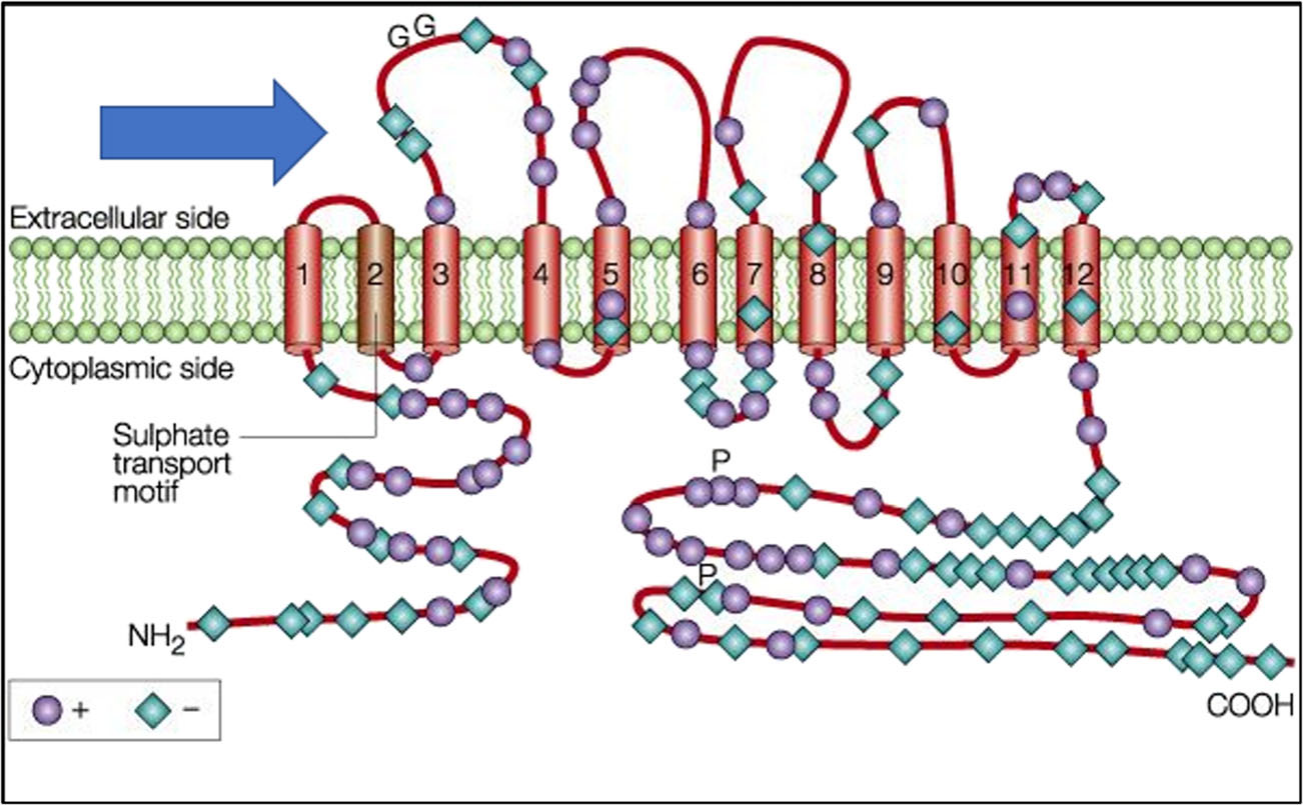
Example of Protein Biomarker. Prestin is an OHC-specific protein that is the prototype biomarker for targeted drug delivery: it only exists on the OHCs, has an extracellular domain, and there is an available ligand that can specifically target this extracellular domain (arrow). Figured adapted with permission from Springer Nature. Dallos P and Fakler B. Prestin, a new type of motor protein. *Nature Reviews. Molecular Cell Biology*, Vol 3, 104-111, 2002

A biomarker is defined as a biological molecule that can be used as an indicator of disease state. This concept can be applied more specifically in the study of acquired diseases of the cochlea to indicate and identify site-specific insults to the structures within the cochlea. In theory, each of the various cell types within the cochlea (IHC, OHC, SV, SG, and supporting cells) has specific molecules (biomarkers—typically proteins) that differentiate it from other cells. Further, many of these acquired cochlear insults cause preferential injury to specific sites within the cochlea (e.g., cisplatin injury to OHC [7], noise-toxicity to spiral ganglion cells in cochlear synaptopathy [8], loop diuretics to the stria vascularis [9]). As such, the concept of cochlear biomarkers opens the potential for targeted, cell-specific therapies in the setting of acquired cochlear injuries. While this concept holds great potential to introduce targeted drug delivery to the inner ear, one of the necessary requirements is that the biomarker target has to be accessible for interaction. From a structural standpoint, this suggests that the biomarker has to be located within the cell’s outer membrane with an available extracellular domain for specific interactions with the treatment or delivery vehicle of interest (Fig. 1). Intracellular targets are theoretically available but would possess limited specificity due to potential non-specific entry into the cell. The ideal components necessary for targeted inner ear drug delivery are listed in Table 1.

**Table 1:** Components of an ideal biomarker for targeted drug delivery

	Ideal situation	Challenges
Accessibility	1. Biomarker target is transmembrane protein with an easily accessible extracellular domain 2. Targeted therapy is delivered prior to or shortly after induced damage 3. Targeted therapy has properties that allow it to enter the inner ear	1. Many known biomarkers and cellular targets are intracellular, limiting specificity and accessibility for therapeutic use 2. Delivering therapeutics to the target biomarker prior to significant cellular damage 3. Many therapies have properties that restrict their entrance to the privileged site of the inner ear [10]
Biomarker/ligand interaction	1. Biomarker protein has a characterized ligand-binding domain 2. Ligands are available that bind to biomarker target	1. Few biomarker targets have well-characterized ligand-binding domains 2. Few compounds are available that bind efficiently to the available domains within biomarker 3. Many of the potential biomarkers are essential for cochlear function. Ligand-protein interaction may alter cellular function
Biomarker specificity	1. Biomarker targeting is specific to a single cell type of interest within the cochlea (e.g., OHC-specific biomarker; stria-specific biomarker; spiral ganglion-specific biomarker)	1. Many of the potential targets available are non-specific and exist in various cell types within the cochlea [11]
Analyzability	1. Quantifiable analysis of biomarker/ligand interaction is available that would provide information about the efficiency of therapeutic delivery	1. Few techniques are available that offer analysis of drug delivery in the clinical setting, and are limited to animal models [12] 2. Clinical outcomes with audiometry are variable and may not reflect what is happening at the cellular level [13]

In the clinical setting, acquired cochlear insults induce permanent, irreversible SNHL. Unfortunately, by the time these disorders are recognized, subjects have already sustained some cochlear damage. Thus, prevention and early recognition are the only options for avoiding insult. If the disorder is identified in a timely manner, oral or intratympanic (IT) therapies become available, although therapy to reverse these acquired insults is currently limited to corticosteroids [14]. Even if treatment is initiated, the outcomes are highly variable. Two factors that would likely improve preservation of cochlear structures in the setting of acquired insults are (1) rapid initiation of therapies (or pre-treatment in the context of known ototoxic medication administration) and (2) targeted, specific delivery of therapies to inner ear structures. If both factors can be addressed, there is significant potential to improve therapeutic efficiency and hearing outcomes in the clinical setting. The timing of the initiation of therapy is dependent upon patient and clinician recognition of the problem and will not be discussed in this review. Targeted delivery of drugs to specific locations within the cochlea is achievable because of the characterization of potential cell-specific biomarkers, and thus will be the focus of this manuscript.

At this time, there is significant translational animal research exploring the concept of targeted local delivery for cochlear disorders [10, 15]; however, there are no targeted therapies available for treatment of the inner ear in the clinical setting. In this review, we aim to discuss potential inner ear biomarkers that may serve to facilitate more efficient mechanisms of inner ear drug delivery. We will discuss potential biomarkers by cell type and provide context as to which clinical disorder may be addressed by targeted therapeutics to each specific cell type. Furthermore, brief information regarding whether potential ligands are available for targeted binding to the biomarker of interest will be provided. Ultimately, biomarker-ligand interactions have the scientific and clinical potential that in the future may lead to therapeutic options for acquired cochlear insults. While we aim to be comprehensive, limited information is available on many of the potential biomarkers; thus, we will detail specifics where possible, and discuss potential utility in other instances.

Biomarkers of congenital cochlear disorders will not be included in this review as this is outside the scope of the discussion. Targeted therapeutics in the management of congenital hearing loss (e.g., Connexin-26, GJB2, related hearing loss) are not currently available because subject are often deaf at birth, and it is challenging to administer therapy in a timely manner. Therapeutic agents for acquired cochlear disorders will not be specifically discussed, as the focus will be on the biomarkers that offer potential targets for therapeutic delivery. It is our hope that this review provides the most up-to-date information regarding the concept of cochlear biomarkers and their role as potential targets for specific delivery of drugs to the inner ear. Emphasis will be placed on the significant translational potential for this research.

## Methodology for literature search

A literature search was performed using the PubMed database. Keywords incorporating each specific cell type of the cochlea were included in our initial search. The cell types we searched were “inner ear,” “outer hair cells,” “inner hair cells,” “spiral ganglion,” and “stria vascularis.” These cochlear cell types were searched with various combinations of the words: “protein,” “target,” and “biomarker.”

Each of the titles and abstracts for these search queries was reviewed by one of the authors. Papers that included proteins primarily responsible for congenital or genetic types of SNHL were excluded from this review. Additionally, any abstracts that were repeated during the search were only included once. The Uniprot (https://www.uniprot.org/) and human protein atlas (www.hprd.org/) databases were then used to determine whether the protein was exposed to the extracellular environment. Papers that had information regarding specific proteins within each cell type were evaluated further to determine whether the protein of interest had been fully characterized. It was noted that many of the proteins identified are found in more than one cell types within the inner ear. In this case, the proteins were characterized, nonetheless, because of their potential utility and availability as biomarkers for targeted drug delivery. In cases where the proteins and genes have more than one name, the name listed on the two protein websites will be used in this review.

**Fig. 2:**
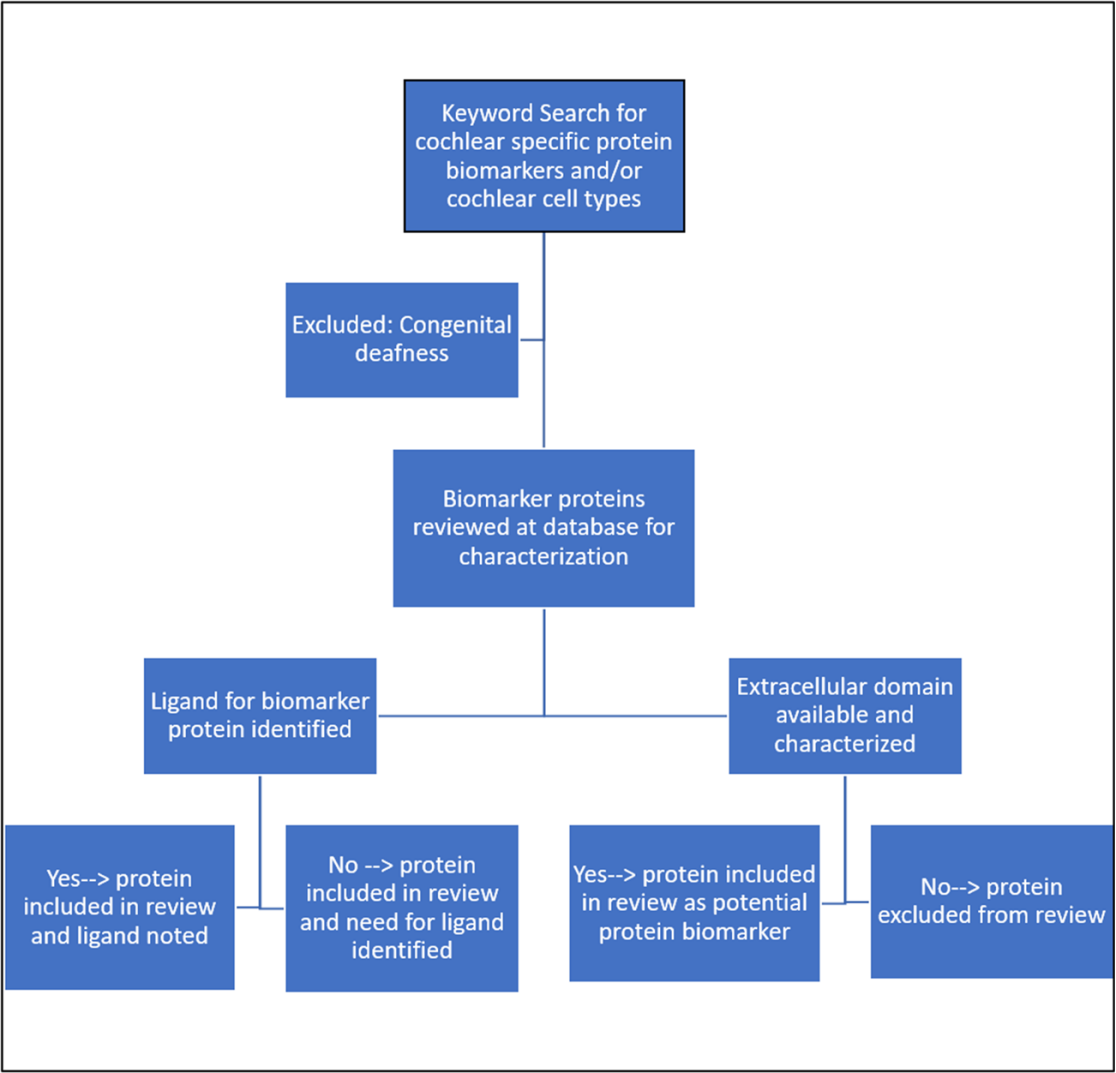
Methods for identifying protein biomarkers available for targeted-drug delivery to the inner ear

Following evaluation within the protein databases, a search for ligand-binding domains and ligands available for each protein was performed (Fig. 2). If a protein was known to have available ligands, they were evaluated for their potential utility to target the drug delivery vehicles to the biomarkers of interest.

## Results

### Inner and outer hair cells

Inner and outer hair cells are among the most vulnerable cell types within the inner ear and are often the primarily damaged cell type in the setting of insult. Despite their vulnerability, they are well characterized both molecularly and functionally, thus offering the realistic potential for specific, targeted delivery to potential hair cell biomarkers. Molecularly, there are a variety of transmembrane protein channels that meet criteria for biomarkers. Functionally, OHCs are largely supportive in function, while IHCs are responsible for signal transduction that converts sound to an electrical signal via its interactions with the cochlear nerve. We will detail some of the well-characterized proteins within the inner and outer hair cells that have significant potential as biomarkers. Additional potential biomarkers are listed in Table 2.

**Table 2:** Potential hair cell-specific biomarker proteins

Protein	Gene	Cell type	Function	Targeting	Ref
Chloride intracellular channel protein 5	CLIC5	OHC, IHC	Channel	None	[16]
Gaba receptors	GABBR1, GABBR2	OHC, IHC	Receptors	None	[17]
Large conductance voltage and calcium activated potassium (BK) channel	KCNMA1	OHC, IHC	Channel	Peptide	[18, 19]
LHFPL tetraspan subfamily member 5 protein	LHFPL5	OHC, IHC	Structural protein	None	[20, 21]
Mechanoelectrical transducer (MET) channel	Various	OHC, IHC	Channel	None	[22]
Otoferlin	OTOF	IHC	Synapse protein	None	[23]
Phospholipid-transporting ATPase IC	ATP8B1	OHC, IHC,	Transport protein	None	[24]
Prestin	SLC26A5	OHC	Motor protein	Peptide	[25–27]
Transmembrane channel-like protein 1	TMC1	OHC, IHC	Channel	None	[28, 29]
Transmembrane channel-like protein 2	TMC2	OHC, IHC	Channel	None	[28]
Vesicular glutamate transporter 3	SLC17A8	IHC, SG	Transport protein	None	[30, 31]

### Hair cell-specific protein biomarkers

#### Prestin

Prestin is a well-described outer hair cell protein that functions as a cochlear amplifier [32]. Unlike many of the other cochlear biomarkers, prestin is specific to the OHCs and does not exist within any other cell type within the cochlea [25]. This makes it particularly appealing as a biomarker for acquired disorders of the OHCs such as cisplatin ototoxicity which preferentially induces damage to the OHCs.

Currently, research is underway investigating targeted delivery of therapies to the OHCs via prestin. This work began after phage display experiments produced a series of peptides that bound to prestin. These peptides were successfully used in the targeted binding of polymersomes to rat cochlear explants [26]. One of the peptides has subsequently been used in vivo to deliver targeted liposomes containing a JNK inhibitor to outer hair cells in a murine model undergoing a severe acoustic insult. The use of the prestin biomarker enabled the payload to be successfully targeted to the outer hair cells and greatly enhanced the protection of the mice’s hearing from the insult [27].

Unfortunately, prestin expression in outer hair cells is downregulated in cells undergoing kanamycin-induced apoptosis. This may limit its usefulness in the treatment of aminoglycoside-induced apoptosis in animal models if therapy is not introduced early enough in the clinical setting [33].

#### Large conductance voltage and calcium-activated potassium (BK) channels

These protein channels are located in the outer membranes of inner and outer hair cells. These channels play an important role in processing the neural feedback from the brain [18]. Clinically, the BK channel has been investigated as a potential channel involved in a tinnitus pathway [19].

A 9-amino acid peptide has been identified which binds tightly to BK channels [19] and can be used both to modulate BK channel activity and potentially as a targeting peptide. Collectively, this work provides the foundation for the concept of BK channels as a potential biomarker available for targeted delivery of therapies aimed at addressing tinnitus.

### Spiral ganglion-specific protein biomarkers

The spiral ganglion cells and neural tissue within the cochlea are an important site for targeted drug delivery because many acquired cochlear disorders have a primary or secondary effect at this location. Synaptopathy of the neuronal elements within the cochlea is a rapidly growing topic within acoustic research [34], and clinically presentation is variable depending on the severity of damage. The few, well-characterized specific protein biomarker targets within the spiral ganglion cells will be discussed, and the remaining will be listed in Table 3.

**Table 3:** Potential spiral ganglion-specific protein biomarkers

Protein	Gene	Cell type	Function	Targeting	Ref
BDNF/NT-3 growth factors receptor	NTRK2	SG	Receptor	Peptide	[35, 36]
NMDA receptors	Various	IHC ribbon synapses	Synapse protein	Peptide	[37, 38]
Substance-P receptor	TACR1	SG	Receptor	Peptide	[39, 40]
Trisialoganglioside clostridial toxin receptor	Not Listed	SG	Receptor	Peptide	[41, 42]

#### Trisialoganglioside clostridial toxin receptor

This trisialoganglioside clostridial toxin receptor (GT1b) is expressed by a large number of neuronal cells within the central and peripheral nervous system. Within the cochlea, it is a receptor located in the spiral ganglion neurons [43, 44]. It serves as a potential prototype biomarker for targeted delivery to spiral ganglion cells due to its specificity to this cell type within the cochlea. Additionally, phage display has identified the Tet1 peptide that binds specifically to the receptor [41]. The advantage of this synthetic peptide is that binding to the trisialoganglioside clostridial toxin receptor does not alter cell signaling within the spiral ganglion. The Tet 1 peptide has been conjugated to polymersomes and delivered to the inner ear using a cochleostomy. The targeted polymersomes bound to the auditory nerve and also to, or adjacent to, the spiral ganglion’s NF-200-positive nerve fibers [42]. Clinically, targeted delivery of therapies to this receptor as a biomarker may provide treatment options for neural-based disorders such as cochlear synaptopathy [4] in the setting of noise exposure. Consequently, the Tet1 peptide is currently the most promising technique to deliver therapeutic payloads to the spiral ganglion or auditory nerve.

#### BDNF/NT-3 growth factors receptor (TrkB)

Within the cochlea, BDNF/NT-3 growth factors receptor is another spiral ganglion-specific biomarker protein for which local delivery of targeted therapies offer potential utility. As with the trisialoganglioside clostridial toxin receptor, phage display has been used to develop binding peptides for these receptors. Recent work has demonstrated that nanoparticles targeted with the TrkB binding A371 peptide bind preferentially to the spiral ganglion’s neuronal cells [35], and that this targeting peptide has enabled Rolipram, a phosphodiesterase inhibitor, to be delivered to the mouse spiral ganglia as a potential agent that improves cell survival [35, 45]. Additional work has suggested the possibility of targeted delivery of the TrkB agonist, 7,8-dihydroxyflavone (DHF) as a means for increasing neurite growth in the setting of kainic-acid cochlear damage [46]. Similarly, targeted delivery of neural growth factors such as BDNF [47] may offer the potential for neural regeneration.

#### *N*-methyl-d-aspartate receptors

These excitatory glutamate receptors are found in a wide range of neural cells that are responsible for opening ion channels and neural signaling. However, in the inner ear, they are found within the synapses of the inner hair cells and thought to play a role in the development of tinnitus [37]. There is significant potential for these receptors as biomarkers for neural tissues at the level of the synapse, and clinical work has already led to the identification of the therapeutic peptide Rapastinel (formally Glyx-13) as a potential therapy for tinnitus [38]. Additional work in animal models has demonstrated ribbon synapse protection from IT application of *N*-methyl-d-aspartate (NMDA) antagonists [37].

### Stria vascularis-specific protein biomarkers

#### Na, K-ATPase

This is a transmembrane protein expressed by a wide range of organs. However, in the cochlea, it is expressed principally in the stria vascularis. The function of Na, K-ATPase in the inner ear is to pump potassium ions into the endolymph to maintain the high potassium concentration within the fluid and the endocochlear potential. In other organs, the pNaKtide peptide has demonstrated binding to the Na, K-ATPase, making it highly likely that the peptide will bind to Na, K-ATPase in the stria vascularis [48]. Interestingly, some research has suggested that this pNaKtide does not alter downstream activity [49]. The role of the stria vascularis in maintaining endocochlear potential is vital to the function of the cochlea, and damage to the stria has been implicated in a variety of acquired disorders. Classically, ototoxicity induced by loop diuretics affects the stria [50] and disrupts the endocochlear potential. Newer evidence supports the concept that cisplatin may impact the stria [51], although the mechanism of cisplatin’s effect here is not yet understood. Clinically, therapies targeted at stria vascularis-specific biomarkers may offer options for prevention of ototoxicity induced by loop diuretics and/or cisplatin.

In addition to the Na, K-ATPase, the *Na*, *K*, *Cl Co*-*Transporter*, and *Barttin* proteins are specific to the stria vascularis and may potentially serve as biomarkers, although targeting peptides are currently not available (Table 4).

**Table 4:** Potential stria vascularis-specific protein biomarkers

Protein	Gene	Cell type	Function	Targeting	Ref
Barttin	BSND	SV	Ion channel subunit	None	[52, 53]
Na, K-ATPase	Multiple Genes	SV	channel	Peptide	[48, 54]
Na, K, Cl Co-transporter	NKCC1	SV	Transport protein	None	[55, 56]

### Non-specific protein biomarkers

Ideally, a biomarker for targeted delivery of therapies should be specific to a particular cell type and location within the cochlea. Nonetheless, the number of protein biomarkers expressed in more than one cell type exceeds the number of cell-specific protein biomarkers. Although non-specific proteins may not be as useful as the cell-specific biomarkers, they can play important roles in the delivery of therapeutics to the inner ear. For example, these proteins can be useful to enhance the delivery of materials to the appropriate inner ear sites in instances where multiple cell types have suffered an insult. Alternatively, they may offer non-specific delivery of therapies to the inner ear cells for which there is currently not a targeting peptide and in scenarios where some delivery is more advantageous than no delivery at all (Table 5). Several non-specific biomarkers are briefly described below.

**Table 5:** Potential non-specific protein biomarkers

Protein	Gene	Cell type	Function	Targeting	Ref
Aquaporin 4	AQP4	Deiters’ and Hansen’s cells	Channel	None	[57]
ATP-sensitive inward rectifier K channel 10	KCNJ10	SV, SG Deiters’	Channel	None	[58, 59]
Basigin	BSG	SV, auditory nerve	Maintenance protein	None	[60]
CD44 antigen	CD44	Outer pillar and Claudius cells	Hyaluronic acid receptor	None	[61]
Clarin 1	CLRN1	OHC, IHC ,SG	Structural protein	None	[62, 63]
Fibroblast growth factor receptor 3	FGFR3	OHC, pillar and Deiters’ Cells	Growth factor	None	[64]
High affinity nerve growth factor receptor	NTRK1	OHC, IHC , SG supporting cell	Receptors	Peptide	[65, 66]
L-type calcium channel	CACNA1C CACNB1	Ubiquitous	Calcium channel	Peptides, Small Molecules	[67–69]
Otoancorin	OTOA	Spiral limbus, tectorial membrane,	Anchoring protein	None	[70]
Pendrin	SLC26A4	Sulcus, spiral prominence	Ion transport	None	[71, 72]
Synaptophysin	SYP	OHC, IHC, SG	Synapse protein	None	[73]
T-type calcium channel	CACNA1H CACNA1G CACNA1I	Ubiquitous	Calcium channel	Peptides, Small Molecules	[67, 74]
Transient receptor potential cation channel subfamily V member 4	TRPV4	SG,SV Support cells	Calcium channel	Small molecules	[75–77]
Type II transmembrane serine protease	HPN	SG,SV	Protease	Small molecule	[78–80]
Vang-like protein 2	VANGL2	OHC, IHC, supporting cells	Anchoring protein	None	[81]
Zinc transporter ZIP8	SLC39A8	Mainly OHC, IHC, SV, supporting cells	Transport protein	None	[82]
Zinc transporter ZIP14	SLC39A14	Mainly OHC, IHC, SV, supporting cells	Transport protein	None	[82]

#### Calcium channels

Calcium channels are ubiquitous within the various cochlear cells. The ability to limit cellular calcium uptake in cochlear cells that have recently experienced an insult has the potential to reduce the number of cells that undergo apoptosis and consequently lessen the hearing deficit induced by the insult [83–85]. L- and T-type calcium channels are the two major forms of calcium channels within the cell [67]. Several natural peptides have been identified that target these calcium channels [68, 74]. In addition to these natural peptides, there is also a range of synthetic calcium channel binding compounds that have been developed by the pharmaceutical industry that may be used as targeting compounds.

#### Neural growth factor receptors

The tyrosine kinase receptor A (TrkA) (gene NTRK1) is a receptor involved in signaling responsible for neural growth and differentiation. Although it is similar in function to TrkB, TrkB is activated by BDNF and is specific to neural tissue, while TrkA is activated by neural growth factor (NGF), and the TrkA receptor is located throughout various cell types within the organ of Corti [65]. The non-specific location of this receptor suggests that the NGF ligand exerts its neurotrophic effects at sites outside of the neural tissue. An additional advantage of this receptor is that peptide fragments of the ligand have been demonstrated to bind to the receptor and could be used to target the protein [66].

#### Serine protease proteins

Type II transmembrane serine protease (TMPRSS1/hepsin) (Gene HPN) is expressed in the spiral ganglion and the stria vascularis [78]. Outside the cochlea, the protein acts as a prostate cancer biomarker, and small molecules that bind to the proteins have been identified. These compounds could be used to target the spiral ganglion and the stria vascularis [79].

## Discussion

There are a wide range of cochlear insults that present with permanent SNHL, tinnitus, and/or vertigo. Many of the insults that result in damage have location-specific sites of injury within the cochlea. Despite the fact that many of the locations and mechanisms of these insults have been established, clinical medicine has been unable to reliably translate this work to specifically target therapies to these sites of injury. In this review, we present an overview of potential cell-specific biomarker proteins within the cochlea as a way for scientists and clinicians to logically think about targeted inner ear delivery. Our goal with this review was to offer a more specific thought process and approach to addressing acquired forms of inner ear damage by suggesting available protein targets for therapeutic delivery to each of the various cell types within the cochlea.

The concept of targeted, location-specific delivery and biomarkers for each of the cell types within the cochlea is a challenging order for scientists and clinicians to address. The privileged nature of the cochlea and its sensitivity to minor changes in the local environment create a logistical challenge of delivering therapies to the correct location without inducing more damage. It is likely necessary to optimize local delivery of therapeutics to the cochlear cells using a variety of techniques that include intratympanic (IT) delivery [86], direct round window membrane (RWM) delivery [27], and intracochlear delivery [87]. Clinically, only IT delivery is routinely used; however, the efficiency of this technique varies based on a number of drug- and patient-related factors. There has been an explosion of research evaluating novel vehicles for therapeutic delivery to the inner ear such as liposomes, hydrogels, and nanoparticles [27, 88–90], and ultimately, identifying the optimal delivery vehicle will improve the yield on our ability to get targeted therapies to the specific biomarkers of interest.

Once a delivery vehicle is optimized, ligands that specifically bind to the biomarker proteins of interest need to be developed. Some of the potential biomarkers within the various cell types have available ligands (see tables); however, many do not. It is reasonable to predict that the next revolution in inner ear drug delivery research may be focused on the production of ligands for proteins that are specific to the various cell types within the cochlea. The clinical implications of cell-specific ligands for targeted delivery of therapies to the inner ear cannot be understated. Currently, non-specific therapies such as corticosteroids are the only available clinical treatment in use to address otologic insults. The mechanism of corticosteroid activity within the inner ear is complex [86, 91, 92] and likely has non-specific interactions that are beyond what we can currently measure. If ligand specificity is developed and refined, this concept would offer the potential to treat various forms of otologic insult and hearing loss in a specific manner. This would not only improve the efficiency of drug delivery, but also provide the potential to measure the amount of therapy delivered more accurately. For example, cisplatin reliably induces insult to the OHC [93]. A ligand that binds specifically to an available OHC biomarker would permit delivery of therapy where it is needed most. In the laboratory setting, techniques would then potentially become available to measure ligand binding interactions, which, in turn, would improve our ability to measure the amount of therapy entering the OHCs. This concept could just as easily be applied to the spiral ganglion cells in the treatment of various causes of cochlear synaptopathy and stria vascularis for loop diuretics.

It warrants mentioning that this review has focused on the use of peptides to target the biomarkers rather than pharmaceutical products (e.g., FDA-approved therapies, commercially available drugs). While pharmaceutical products can bind to the biomarkers as tightly and with the same specificity as peptides, they can be potentially much more challenging to conjugate to nanoparticles containing the appropriate therapeutic payload. The methodology to conjugate peptides to the nanoparticle’s coat is well-established and relatively straightforward to perform [94]. Although the methodology to conjugate pharmaceutical compounds to nanoparticles is possible, it is not as widely used, and largely limited to applications in oncology [95].

The simplicity of the concept of targeted delivery to cell-specific biomarkers is currently overshadowed by the complexity of execution. There are layers of complexity to each step of this process that necessitate discussion. From a clinical perspective, it is a risk to initiate treatment on subjects who have any residual cochlear function because the cochlear cells are particularly sensitive to changes within the local environment [96, 97]. Thus, many subjects with acquired forms of otologic insult and some remaining natural hearing are unlikely to be candidates in clinical trials for the initiation of targeted treatments [98]. Next, finding the appropriate combination of delivery vehicle, ligand, and therapy can be an overwhelming task. While there are a handful of vehicles for drug delivery (i.e., hydrogels, liposomes, etc.), the number of potential ligands is extensive when both natural and synthetic ligands are accounted for. Even if the correct combination of vehicle and ligand is discovered, there is potential that conjugating the vehicle, ligand, and therapy may compromise the function or binding efficiency to the target. Similarly, science has not achieved reliable success in determining therapies that preserve cochlear function or reverse cochlear insult. Thus, it seems that the application of these concepts is not as close as many think. Nonetheless, there is potential to apply some of these concepts to hearing preservation during the introduction of a cochlear implant (CI) [99]. As indications for CI expand to include subjects with residual hearing, it offers a realistic opportunity to introduce these concepts to subjects that have little cochlear reserve, and thus, little to lose. In fact, some research is already evaluating the role of BDNF and cochlear implant for SGN survival [100, 101].

In addition to the challenges listed above, the physiological response to cellular insult introduces additional challenges. The first challenge is that response to insult and delivery of therapy is time sensitive. For the treating clinician, this represents a logistical challenge of getting subjects treated before irreversible damage develops. This typically occurs within the first few days after insult [102]. Similarly, cells within the cochlea respond to insult by altering their homeostasis, which manifests as a change in protein production and function. For example, prestin expression in outer hair cells is downregulated in cells undergoing kanamycin-induced apoptosis [33]. Thus, despite the theoretical utility of prestin as an OHC-specific protein biomarker, its utility may be limited in the setting of aminoglycoside-induced apoptosis in both animal models and in the clinical setting if therapy is not introduced early enough [33]. Alternatively, proteins can be upregulated which may introduce new biomarker targets, as in the setting of cisplatin ototoxicity [103]. These concepts will likely warrant consideration as the concept of biomarker targeting is refined.

Another consideration with this concept is that we are currently only able to present this treatment approach for acquired forms of otologic insults in the setting of normal structure and function of the cochlea prior to insult. Many of the well-characterized forms of hearing loss are congenital or present at birth [104], which may eliminate the potential for non-gene-based therapies to reverse the process. Fortunately, there is currently a significant amount of animal research being undertaken that explores treatment and potential therapies of congenital and syndromic forms of hearing loss [105, 106]. Future considerations should aim at targeting specific sites of dysfunction in these genetic forms of hearing loss, particularly as gene therapies become available [107].

Ideally, the binding of the targeting peptide to its biomarker should not affect the biomarker’s cellular function. However, as the peptides are expected to target outer membrane proteins that are exposed to the extracellular environment, it is highly likely that many of the targeted biomarkers will be receptors or channel proteins. In such cases, the targeting peptides may act as an agonist or antagonist modulating cellular function [108]. For instance, care has to be taken employing the pNaKtide peptide to target the stria vascularis as the protein acts as an agonist modifying Na/K-ATPase-mediated amplification of ROS signaling [48, 109]. This concern of altered cellular function has previously been demonstrated where analogs of NGF bind TrkA and inhibit neurite outgrowth [66]. Refining and understanding ligand-target interactions will be necessary to avoid these unwanted interactions as progress is made.

This review has identified a large number of proteins that have the potential to act as biomarkers. Unfortunately, ligands and targeting peptides are not available for the majority of these proteins. This is an indication that the study of targeted delivery of therapeutic material to inner ear cells is in its infancy, and the future for targeted nanoparticles to deliver therapies to the inner ear holds great potential. As more work is accomplished, this technology should also enable genetic material to be delivered to cells in order to treat patients with genetically induced progressive hearing loss in addition to the acquired forms of hearing loss that we discuss.

**Fig. 3:**
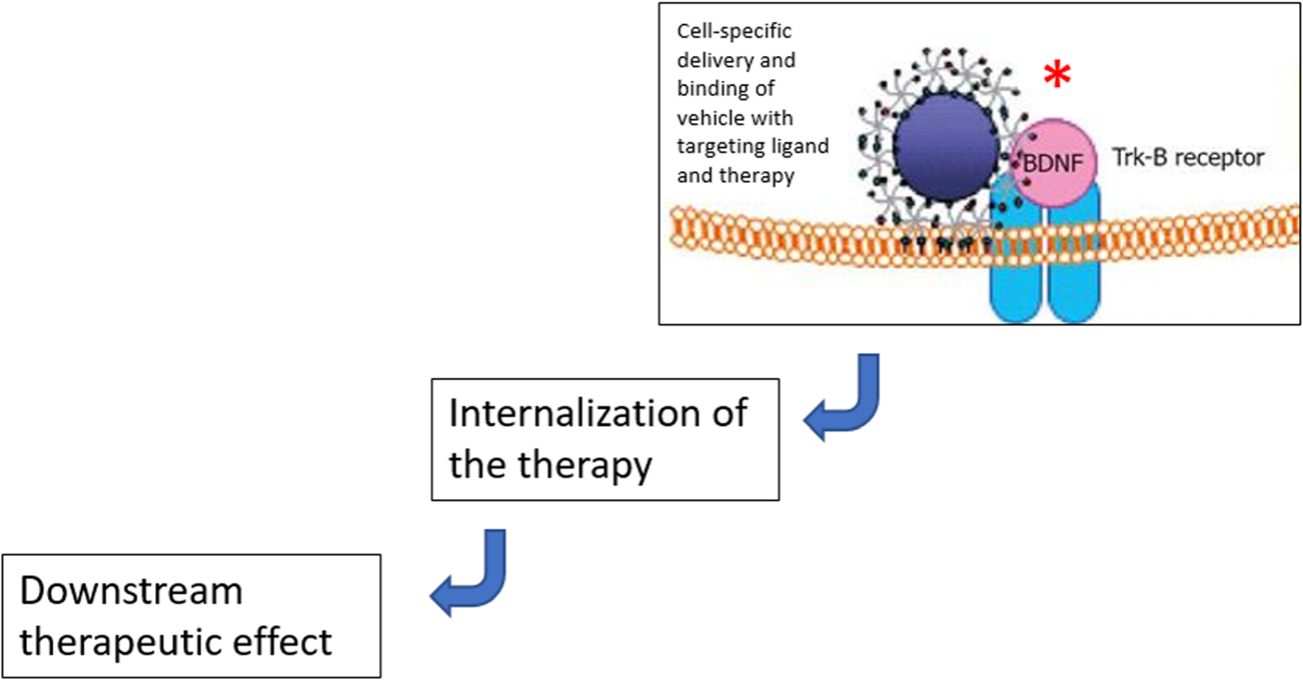
Conceptual approach to cell-specific targeted therapeutics delivery in treatment of cochlea-related diseases. The criteria necessary for targeted therapeutic delivery are represented in this image (see “Discussion” section). The *TrkB* receptor is specific to spiral ganglion tissue. The receptor has an accessible extracellular domain that allows for interaction with a targeted ligand (asterisk) and downstream therapeutic effects. Figure adapted with permission under the terms of the Creative Commons Attribution-Noncommercial (CC BY-NC 4.0) License. Pyykkö I, Zou J, Zhang Y, Zhang W, Feng H, Kinnunen P. Nanoparticle based inner ear therapy. *World J Otorhinolaryngol* 2013; 3(4): 114-133

Overall, cell-specific targeting for inner ear drug delivery would fulfill the following criteria: (1) a protein biomarker that is specific to a single cell type (IHC, OHC, SV, SG, supporting cells) should be available; (2) this specific biomarker should have an accessible extracellular domain; (3) a ligand that interacts with the cell-specific biomarker protein should be available; and (4) the ligand-protein interaction should lead to internalization of the therapy for downstream action. These ideal characteristics are summarized in Fig. 3. Despite the challenges that lie ahead in the evolution of targeted delivery of therapies for acquired inner ear disorders, progress is occurring. Our understanding of the function and structure of the cells within the inner ear has evolved tremendously within the last couple of decades. Biomolecular research has elucidated the function of many of the proteins that we present here as biomarkers and targets for therapeutic delivery. It is conceivable that ligand discovery is not far behind. The essential component to all of this work will be the collaborative efforts on the part of clinicians and scientists to make this concept a reality. Clinicians need to remain unsatisfied with the status quo of otologic therapies, and scientists need to continue to explore applications of their work to wide-spread burden of otologic disorders. This idea presents an opportunity to apply the discoveries of basic science to the treatment of otologic disease. Importantly, it also represents an opportunity to begin the next phase of inner ear therapeutics that addresses the shortcoming of previous generations of treatments and combines targeted, specific technology as medicine moves into an era of personalized medicine [110].

## References

[1] Wang J, Powers NL, Hofstetter P, Trautwein P, Ding D, Salvi R (1997). Effects of selective inner hair cell loss on auditory nerve fiber threshold, tuning and spontaneous and driven discharge rate. Hear Res.

[2] Marcotti W, van Netten SM, Kros CJ (2005). The aminoglycoside antibiotic dihydrostreptomycin rapidly enters mouse outer hair cells through the mechano-electrical transducer channels. J Physiol.

[3] Ikeda K, Oshima T, Hidaka H (1997). Molecular and clinical implications of loop diuretic ototoxicity. Hear Res.

[4] Liberman LD, Suzuki J, Liberman MC (2015). Dynamics of cochlear synaptopathy after acoustic overexposure. Journal of the Association for Research in Otolaryngology : JARO.

[5] Wan G, Corfas G, Stone JS (2013). Inner ear supporting cells: rethinking the silent majority. Semin Cell Dev Biol.

[6] McCall AA, Swan EE, Borenstein JT (2010). Drug delivery for treatment of inner ear disease: current state of knowledge. Ear Hear.

[7] Rybak LP, Whitworth CA, Mukherjea D, Ramkumar V (2007). Mechanisms of cisplatin-induced ototoxicity and prevention. Hear Res.

[8] Kurabi A, Keithley EM, Housley GD, Ryan AF, Wong AC (2017). Cellular mechanisms of noise-induced hearing loss. Hear Res.

[9] Rybak LP (1993). Ototoxicity of loop diuretics. Otolaryngol Clin N Am.

[10] Hao J, Li SK (2019). Inner ear drug delivery: recent advances, challenges, and perspective. Eur J Pharm Sci.

[11] Li L, Chao T, Brant J, O’Malley B Jr, Tsourkas A, Li D (2017). Advances in nano-based inner ear delivery systems for the treatment of sensorineural hearing loss. Adv Drug Deliv Rev.

[12] Salt AN, Plontke SK (2018). Pharmacokinetic principles in the inner ear: Influence of drug properties on intratympanic applications. Hear Res.

[13] Landegger LD, Psaltis D, Stankovic KM (2016). Human audiometric thresholds do not predict specific cellular damage in the inner ear. Hear Res.

[14] Rauch SD, Halpin CF, Antonelli PJ (2011). Oral vs intratympanic corticosteroid therapy for idiopathic sudden sensorineural hearing loss: a randomized trial. JAMA.

[15] Abi-Hachem RN, Zine A, Van De Water TR (2010). The injured cochlea as a target for inflammatory processes, initiation of cell death pathways and application of related otoprotectives strategies. Recent Pat CNS Drug Discov.

[16] Gagnon LH, Longo-Guess CM, Berryman M (2006). The chloride intracellular channel protein CLIC5 is expressed at high levels in hair cell stereocilia and is essential for normal inner ear function. J Neurosci.

[17] Wedemeyer C, de San Martin JZ, Ballestero J (2013). Activation of presynaptic GABA(B(1a,2)) receptors inhibits synaptic transmission at mammalian inhibitory cholinergic olivocochlear-hair cell synapses. J Neurosci.

[18] Ruttiger L, Sausbier M, Zimmermann U (2004). Deletion of the Ca2+-activated potassium (BK) alpha-subunit but not the BKbeta1-subunit leads to progressive hearing loss. Proc Natl Acad Sci U S A.

[19] Scott LL, Brecht EJ, Philpo A (2017). A novel BK channel-targeted peptide suppresses sound evoked activity in the mouse inferior colliculus. Sci Rep.

[20] Xiong W, Grillet N, Elledge HM, Wagner TF, Zhao B, Johnson KR, Kazmierczak P, Müller U (2012). TMHS is an integral component of the mechanotransduction machinery of cochlear hair cells. Cell.

[21] Gyorgy B, Sage C, Indzhykulian AA (2017). Rescue of hearing by gene delivery to inner-ear hair cells using exosome-associated AAV. Mol Ther.

[22] Fettiplace R (2009). Defining features of the hair cell mechanoelectrical transducer channel. Pflugers Arch - Eur J Physiol.

[23] Pangrsic T, Reisinger E, Moser T (2012). Otoferlin: a multi-C2 domain protein essential for hearing. Trends Neurosci.

[24] Stapelbroek JM, Peters TA, van Beurden DHA (2009). ATP8B1 is essential for maintaining normal hearing. Proc Natl Acad Sci U S A.

[25] Zheng J, Shen W, He DZ (2000). Prestin is the motor protein of cochlear outer hair cells. Nature.

[26] Surovtseva EV, Johnston AH, Zhang W (2012). Prestin binding peptides as ligands for targeted polymersome mediated drug delivery to outer hair cells in the inner ear. Int J Pharm.

[27] Kayyali MN, Wooltorton JRA, Ramsey AJ, Lin M, Chao TN, Tsourkas A, O'Malley BW Jr, Li D (2018). A novel nanoparticle delivery system for targeted therapy of noise-induced hearing loss. J Control Release.

[28] Pan B, Geleoc GS, Asai Y (2013). TMC1 and TMC2 are components of the mechanotransduction channel in hair cells of the mammalian inner ear. Neuron.

[29] Pan B, Akyuz N, Liu X-P (2018). TMC1 forms the pore of mechanosensory transduction channels in vertebrate inner ear hair cells. Neuron.

[30] Akil O, Seal RP, Burke K, Wang C, Alemi A, During M, Edwards RH, Lustig LR (2012). Restoration of hearing in the VGLUT3 knockout mouse using virally mediated gene therapy. Neuron.

[31] Lee Y, Kim HR, Ahn SC (2015). Vesicular glutamate transporter 3 is strongly upregulated in cochlear inner hair cells and spiral ganglion cells of developing circling mice. Neurosci Lett.

[32] Dallos P, Fakler B (2002). Prestin, a new type of motor protein. Nat Rev Mol Cell Biol.

[33] Yu L, Jiang XH, Zhou Z (2011). A protective mechanism against antibiotic-induced ototoxicity: role of prestin. PloS One.

[34] Kujawa SG, Liberman MC (2015). Synaptopathy in the noise-exposed and aging cochlea: Primary neural degeneration in acquired sensorineural hearing loss. Hear Res.

[35] Glueckert R, Pritz CO, Roy S (2015). Nanoparticle mediated drug delivery of rolipram to tyrosine kinase B positive cells in the inner ear with targeting peptides and agonistic antibodies. Front Aging Neurosci.

[36] Ranjan S, Sood R, Dudas J, Glueckert R, Schrott-Fischer A, Roy S, Pyykkö I, Kinnunen PK (2012). Peptide-mediated targeting of liposomes to TrkB receptor-expressing cells. Int J Nanomedicine.

[37] Bing D, Lee SC, Campanelli D (2015). Cochlear NMDA receptors as a therapeutic target of noise-induced tinnitus. Cell Physiol Biochem.

[38] Moskal JR, Burgdorf JS, Stanton PK (2017). The development of rapastinel (Formerly GLYX-13); A rapid acting and long lasting antidepressant. Curr Neuropharmacol.

[39] Ito K, Rome C, Bouleau Y, Dulon D (2002). Substance P mobilizes intracellular calcium and activates a nonselective cation conductance in rat spiral ganglion neurons. Eur J Neurosci.

[40] Huang S-C, Korlipara VL (2010). Neurokinin-1 receptor antagonists: a comprehensive patent survey. Expert Opin Ther Pat.

[41] Liu JK, Teng Q, Garrity-Moses M, Federici T, Tanase D, Imperiale MJ, Boulis NM (2005). A novel peptide defined through phage display for therapeutic protein and vector neuronal targeting. Neurobiol Dis.

[42] Zhang Y, Zhang W, Johnston AH, Newman TA, Pyykkö I, Zou J (2012). Targeted delivery of Tet1 peptide functionalized polymersomes to the rat cochlear nerve. Int J Nanomedicine.

[43] Santi PA, Mancini P, Barnes C (1994). Identification and localization of the GM1 ganglioside in the cochlea using thin-layer chromatography and cholera toxin. J Histochem Cytochem.

[44] Maguchi S, Gasa S, Matsushima J (1991). Glycolipids in rat cochlea. Auris Nasus Larynx.

[45] Kranz K, Warnecke A, Lenarz T (2014). Phosphodiesterase type 4 inhibitor rolipram improves survival of spiral ganglion neurons in vitro. PLoS One.

[46] Kempfle JS, Nguyen K, Hamadani C (2018). Bisphosphonate-linked TrkB agonist: cochlea-targeted delivery of a neurotrophic agent as a strategy for the treatment of hearing loss. Bioconjug Chem.

[47] Khalin I, Alyautdin R, Kocherga G, Bakar MA (2015). Targeted delivery of brain-derived neurotrophic factor for the treatment of blindness and deafness. Int J Nanomedicine.

[48] Sodhi K, Maxwell K, Yan Y (2015). pNaKtide inhibits Na/K-ATPase reactive oxygen species amplification and attenuates adipogenesis. Sci Adv.

[49] Li Z, Zhang Z, Xie JX (2011). Na/K-ATPase mimetic pNaKtide peptide inhibits the growth of human cancer cells. J Biol Chem.

[50] Rybak LP (1993). Ototoxicity of loop diuretics. Otolaryngol Clin N Am.

[51] Breglio AM, Rusheen AE, Shide ED (2017). Cisplatin is retained in the cochlea indefinitely following chemotherapy. Nat Commun.

[52] Gradogna A, Pusch M (2010). Molecular Pharmacology of Kidney and Inner Ear CLC-K Chloride Channels. Front Pharmacol.

[53] Rickheit G, Maier H, Strenzke N, Andreescu CE, de Zeeuw CI, Muenscher A, Zdebik AA, Jentsch TJ (2008). Endocochlear potential depends on Cl- channels: mechanism underlying deafness in Bartter syndrome IV. EMBO J.

[54] Erichsen S, Stierna P, Bagger-Sjoback D (1998). Distribution of Na,K-ATPase is normal in the inner ear of a mouse with a null mutation of the glucocorticoid receptor. Hear Res.

[55] Crouch JJ, Sakaguchi N, Lytle C, Schulte BA (1997). Immunohistochemical localization of the Na-K-Cl co-transporter (NKCC1) in the gerbil inner ear. J Histochem Cytochem.

[56] Watabe T, Xu M, Watanabe M (2017). Time-controllable Nkcc1 knockdown replicates reversible hearing loss in postnatal mice. Sci Rep.

[57] Mhatre AN, Stern RE, Li J, Lalwani AK (2002). Aquaporin 4 expression in the mammalian inner ear and its role in hearing. Biochem Biophys Res Commun.

[58] Scholl UI, Choi M, Liu T, Ramaekers VT, Häusler MG, Grimmer J, Tobe SW, Farhi A, Nelson-Williams C, Lifton RP (2009). Seizures, sensorineural deafness, ataxia, mental retardation, and electrolyte imbalance (SeSAME syndrome) caused by mutations in KCNJ10. Proc Natl Acad Sci U S A.

[59] Jin Z, Wei D, Jarlebark L (2006). Developmental expression and localization of KCNJ10 K+ channels in the guinea pig inner ear. Neuroreport.

[60] Kawano H, Tono T, Kadomatsu K, Muramatsu T, Komune S (2003). Expression of basigin, a member of the immunoglobulin superfamily, in the mouse cochlea. ORL J Otorhinolaryngol Relat Spec.

[61] Hertzano R, Puligilla C, Chan S-L (2010). CD44 is a marker for the outer pillar cells in the early postnatal mouse inner ear. Journal of the Association for Research in Otolaryngology : JARO.

[62] Geng R, Omar A, Gopal SR (2017). Modeling and preventing progressive hearing loss in usher syndrome III. Sci Rep.

[63] Geng R, Melki S, Chen DHC (2012). The mechanosensory structure of the hair cell requires clarin-1, a protein encoded by Usher syndrome III causative gene. J Neurosci.

[64] Colvin JS, Bohne BA, Harding GW (1996). Skeletal overgrowth and deafness in mice lacking fibroblast growth factor receptor 3. Nat Genet.

[65] Dai CF, Steyger PS, Wang ZM (2004). Expression of Trk a receptors in the mammalian inner ear. Hear Res.

[66] LeSauteur L, Wei L, Gibbs BF (1995). Small peptide mimics of nerve growth factor bind TrkA receptors and affect biological responses. J Biol Chem.

[67] Ceriani F, Mammano F (2012). Calcium signaling in the cochlea—molecular mechanisms and physiopathological implications. Cell Commun Signal.

[68] Findeisen F, Campiglio M, Jo H, Abderemane-Ali F, Rumpf CH, Pope L, Rossen ND, Flucher BE, DeGrado W, Minor DL Jr (2017). Stapled voltage-gated calcium channel (CaV) alpha-interaction domain (AID) peptides act as selective protein-protein interaction inhibitors of CaV function. ACS Chem Neurosci.

[69] Zhang SY, Robertson D, Yates G, Everett A (1999). Role of L-type Ca(2+) channels in transmitter release from mammalian inner hair cells I. Gross sound-evoked potentials. J Neurophysiol.

[70] Zwaenepoel I, Mustapha M, Leibovici M (2002). Otoancorin, an inner ear protein restricted to the interface between the apical surface of sensory epithelia and their overlying acellular gels, is defective in autosomal recessive deafness DFNB22. Proc Natl Acad Sci U S A.

[71] Royaux IE, Belyantseva IA, Wu T, Kachar B, Everett LA, Marcus DC, Green ED (2003). Localization and functional studies of pendrin in the mouse inner ear provide insight about the etiology of deafness in pendred syndrome. J Assoc Res Otolaryngol.

[72] Cremers CW, Admiraal RJ, Huygen PL, Bolder C, Everett LA, Joosten FB, Green ED, van Camp G, Otten BJ (1998). Progressive hearing loss, hypoplasia of the cochlea and widened vestibular aqueducts are very common features in Pendred's syndrome. Int J Pediatr Otorhinolaryngol.

[73] Khalifa SAM, Friberg U, Illing RB (2003). Synaptophysin immunohistochemistry in the human cochlea. Hear Res.

[74] Bladen C, Hamid J, Souza IA (2014). Block of T-type calcium channels by protoxins I and II. Mol Brain.

[75] Lee J-H, Park C, Kim S-J (2013). Different uptake of gentamicin through TRPV1 and TRPV4 channels determines cochlear hair cell vulnerability. Exp Mol Med.

[76] White JPM, Cibelli M, Urban L (2016). TRPV4: molecular conductor of a diverse Orchestra. Physiol Rev.

[77] Shen J, Harada N, Kubo N, Liu B, Mizuno A, Suzuki M, Yamashita T (2006). Functional expression of transient receptor potential vanilloid 4 in the mouse cochlea. Neuroreport.

[78] Guipponi M, Tan J, Cannon PZF (2007). Mice deficient for the type II transmembrane serine protease, TMPRSS1/hepsin, exhibit profound hearing loss. Am J Pathol.

[79] Subedi M, Minn I, Chen J (2016). Design, synthesis and biological evaluation of PSMA/hepsin-targeted heterobivalent ligands. Eur J Med Chem.

[80] Guipponi M, Toh M-Y, Tan J, Park D, Hanson K, Ballana E, Kwong D, Cannon PZ, Wu Q, Gout A, Delorenzi M, Speed TP, Smith RJ, Dahl HH, Petersen M, Teasdale RD, Estivill X, Park WJ, Scott HS (2008). An integrated genetic and functional analysis of the role of type II transmembrane serine proteases (TMPRSSs) in hearing loss. Hum Mutat.

[81] Montcouquiol M, Sans N, Huss D, Kach J, Dickman JD, Forge A, Rachel RA, Copeland NG, Jenkins NA, Bogani D, Murdoch J, Warchol ME, Wenthold RJ, Kelley MW (2006). Asymmetric localization of Vangl2 and Fz3 indicate novel mechanisms for planar cell polarity in mammals. J Neurosci.

[82] Ding D, Salvi R, Roth JA (2014). Cellular localization and developmental changes of Zip8, Zip14 and transferrin receptor 1 in the inner ear of rats. Biometals.

[83] So HS, Park C, Kim HJ, Lee JH, Park SY, Lee JH, Lee ZW, Kim HM, Kalinec F, Lim DJ, Park R (2005). Protective effect of T-type calcium channel blocker flunarizine on cisplatin-induced death of auditory cells. Hear Res.

[84] Cox R, Naples JG, Bonaiuto G, Parham K. Emerging concepts for otoprotection and early detection of cisplatin-induced ototoxicity. 2017.

[85] Sekiya T, Yagihashi A, Asano K, Suzuki S (2002). Nimodipine ameliorates trauma-induced cochlear neuronal death. Neurol Res.

[86] Hargunani CA, Kempton JB, DeGagne JM (2006). Intratympanic injection of dexamethasone: time course of inner ear distribution and conversion to its active form. Otol Neurotol.

[87] Borenstein JT (2011). Intracochlear drug delivery systems. Expert Opin Drug Deliv.

[88] Gyorgy B, Sage C, Indzhykulian AA (2017). Rescue of hearing by gene delivery to inner-ear hair cells using exosome-associated AAV. Mol Ther.

[89] Yoon JY, Yang K-J, Park S-N (2016). The effect of dexamethasone/cell-penetrating peptide nanoparticles on gene delivery for inner ear therapy. Int J Nanomedicine.

[90] Rahmani S, Ross AM, Park T-H, Durmaz H, Dishman AF, Prieskorn DM, Jones N, Altschuler RA, Lahann J (2016). Dual release carriers for cochlear delivery. Adv Healthc Mater.

[91] Trune DR, Canlon B (2012). Corticosteroid therapy for hearing and balance disorders. Anat Record (Hoboken).

[92] Patel M (2017). Intratympanic corticosteroids in Meniere's disease: a mini-review. J Otol.

[93] Rybak LP, Whitworth CA (2005). Ototoxicity: therapeutic opportunities. Drug Discov Today.

[94] Spicer CD, Jumeaux C, Gupta B (2018). Peptide and protein nanoparticle conjugates: versatile platforms for biomedical applications. Chem Soc Rev.

[95] Miele E, Spinelli GP, Miele E, Tomao F, Tomao S (2009). Albumin-bound formulation of paclitaxel (Abraxane ABI-007) in the treatment of breast cancer. Int J Nanomedicine.

[96] Nassiri AM, Yawn RJ, Gifford RH et al. Intraoperative electrically evoked compound action potential (ECAP) measurements in traditional and hearing preservation cochlear implantation. J Am Acad Audiol, 2019. 10.3766/jaaa.18052. 10.3766/jaaa.18052 PMC6999088 31274070

[97] Trune DR (2010). Ion homeostasis in the ear: mechanisms, maladies, and management. Curr Opin Otolaryngol Head Neck Surg.

[98] Safety, Tolerability and Efficacy for CGF166 in Patients With Unilateral or Bilateral Severe-to-profound Hearing Loss. https://clinicaltrials.gov/ct2/show/NCT02132130, vol. Accessed 30 Sept 2019.

[99] Roland JT, Gantz BJ, Waltzman SB (2018). Long-term outcomes of cochlear implantation in patients with high-frequency hearing loss. Laryngoscope.

[100] Rejali D, Lee VA, Abrashkin KA, Humayun N, Swiderski DL, Raphael Y (2007). Cochlear implants and ex vivo BDNF gene therapy protect spiral ganglion neurons. Hear Res.

[101] Leake PA, Hradek GT, Hetherington AM, Stakhovskaya O (2011). Brain-derived neurotrophic factor promotes cochlear spiral ganglion cell survival and function in deafened, developing cats. J Comp Neurol.

[102] Liberman MC (2016). Noise-induced hearing loss: permanent versus temporary threshold shifts and the effects of hair cell versus neuronal degeneration. Adv Exp Med Biol.

[103] Oh G-S, Kim H-J, Choi J-H (2011). Activation of lipopolysaccharide-TLR4 signaling accelerates the ototoxic potential of cisplatin in mice. J Immunol.

[104] Smith RJH, Bale JF, White KR (2005). Sensorineural hearing loss in children. Lancet (London, England).

[105] Pan B, Askew C, Galvin A, Heman-Ackah S, Asai Y, Indzhykulian AA, Jodelka FM, Hastings ML, Lentz JJ, Vandenberghe LH, Holt JR, Géléoc GS (2017). Gene therapy restores auditory and vestibular function in a mouse model of Usher syndrome type 1c. Nat Biotechnol.

[106] Iizuka T, Kamiya K, Gotoh S (2015). Perinatal Gjb2 gene transfer rescues hearing in a mouse model of hereditary deafness. Hum Mol Genet.

[107] Kelly KM, Lalwani AK (2015). On the distant horizon--medical therapy for sensorineural hearing loss. Otolaryngol Clin N Am.

[108] Zhang Y, Zhang W, Johnston AH, Newman TA, Pyykkö I, Zou J (2012). Targeted delivery of Tet1 peptide functionalized polymersomes to the rat cochlear nerve. Int J Nanomedicine.

[109] Liu J, Lilly MN, Shapiro JI (2018). Targeting Na/K-ATPase signaling: a new approach to control oxidative stress. Curr Pharm Des.

[110] Vogenberg FR, Isaacson Barash C, Pursel M (2010). Personalized medicine: part 1: evolution and development into theranostics. P & T : a Peer-Reviewed Journal for Formulary Management.

